# Goodness of fit for the logistic regression model using relative belief

**DOI:** 10.1186/s40488-017-0070-7

**Published:** 2017-08-31

**Authors:** Luai Al-Labadi, Zeynep Baskurt, Michael Evans

**Affiliations:** 1 Department of Statistical Sciences, University of Toronto, 100 St. George St., Toronto, M5S 3G3 Canada; 2 Genetics and Genome Biology, Hospital for Sick Children, 686 Bay Street, Toronto, M5G 0A4 Canada

**Keywords:** Model checking, Concentration, Relative belief ratio, 62F15

## Abstract

A logistic regression model is a specialized model for product-binomial data. When a proper, noninformative prior is placed on the unrestricted model for the product-binomial model, the hypothesis *H*_0_ of a logistic regression model holding can then be assessed by comparing the concentration of the posterior distribution about *H*_0_ with the concentration of the prior about *H*_0_. This comparison is effected via a relative belief ratio, a measure of the evidence that *H*_0_ is true, together with a measure of the strength of the evidence that *H*_0_ is either true or false. This gives an effective goodness of fit test for logistic regression.

## Introduction

Suppose there is a response *Y*∈{0,1} related to *k* predictors (*X*_1_,…,*X*_*k*_) via the logistic regression model *p*(**x** ^′^ **β**)=*P*(*Y*=1 | *X*_1_=*x*_1_,…,*X*_*k*_=*x*_*k*_) where

1 $$ p(\mathbf{x}^{\prime}\mathbf{\beta})=\exp\left\{ \mathbf{x}^{\prime }\mathbf{\beta}\right\} /\left(1+\exp\left\{ \mathbf{x}^{\prime }\mathbf{\beta}\right\} \right).   $$

and **x**=(*x*_1_,…,*x*_*k*_)**,** **β**=(*β*_1_,…,*β*_*k*_)∈*R* ^*k*^. While the use of this model is quite common, the question concerning whether or not the model actually holds has not been fully dealt with in the literature. It is our purpose here to develop a Bayesian approach to this problem.

It is to be noted that, irrespective of whether or not (1) holds, *Y* | *X*_1_=*x*_1_,…,*X*_*k*_=*x*_*k*_∼ Bernoulli (*θ*(**x**)) for some *θ*(**x**)∈ [0,1] and, if a sample of *n*(**x**) is taken at these settings of the predictors, then $s(\mathbf {x})={\sum \nolimits }_{i=1}^{n(\mathbf {x})}Y_{i}\,|\,X_{1}=x_{1},\ldots,X_{k}=x_{k}\sim $ binomial (*n*(**x**),*θ*(**x**)). That such data is indeed binomial (independence and constant probability) can be assessed via a runs test applied to each such subsample when *n*(**x**)>1. With random sampling from a large enough population the binomial assumption is surely approximately correct and so this aspect of possible model failure is ignored here. The question of interest is whether or not *θ*(**x**) is given by (1), at least to a reasonable approximation, and this is the logistic regression assumption.

When there are only categorical predictors, so the *X*_*i*_ correspond to dummy variables, then indeed (1) holds as then only one of the *x*_*i*_=1 with the rest equal to 0 and the relevant probability is exp{*β*_*i*_}/(1+ exp{*β*_*i*_}) where *β*_*i*_ is the log of the odds in favor of 1. So in this case the logistic regression is just a reparameterization of the product-binomial model. Goodness of fit beyond the runs tests is then not relevant.

The case when there is at least some quantitative predictors is thus the one of interest. It is to be noted that in any well-designed study there should always be replication, namely, *n*(**x**)>1 for some of the **x** **,** for precisely the reason that model checking is a necessary part of any statistical analysis in a scientific context. It may be, however, that the data was collected in a somewhat haphazard way, and while model checking is still a requirement, it can’t be expected that this will be as effective as in the designed context.

The general approach taken here can be described as follows. First a noninformative prior is placed on the *θ*(**x**). A specific meaning is applied to the word noninformative here, namely, we require that there is no possibility of there being prior-data conflict. Prior-data conflict loosely means that the prior places the bulk of its mass in a region of the parameter space which the data identifies as being unlikely to contain the true value. Prior-data conflict can be measured in a number of ways and this is discussed in Section 2.

Suppose that there are *m* values for **x** so the full space for the *θ*(**x**) is [0,1]^*m*^. Let *H*_0_⊂ [0,1]^*m*^ be the subset that corresponds to *θ*(**x**)=*p*(**x** ^′^ **β**) for some **β**∈*R* ^*k*^. The prior on the *θ*(**x**) leads to a posterior distribution for these quantities. Intuitively, if the posterior is more concentrated about *H*_0_ than the prior, then this is evidence in favor of *H*_0_ with the opposite holding when the posterior is less concentrated about *H*_0_ than the prior. Once a method of measuring concentration about *H*_0_ is selected, this evidence can be measured via a relative belief ratio. While a relative belief ratio is somewhat like a Bayes factor, it will be seen to differ in some key ways. Furthermore, a measure of the strength of this evidence, whether for or against *H*_0_, is presented. Several natural measures of concentration are considered. This is all discussed in Section 3. In Section 4 aspects of the computations are considered including application to a number of examples.

Tsutukawa and Lin (1986) and Bedrick et al. (1996, 1997) are concerned with the Bayesian analysis of logistic regression models although not with goodness of fit. With a *p*-value based on asympotics, a commonly used goodness of fit statistic for logistic regression is the deviance statistic which is twice the difference between the maximized log-likelihood with no constraints and the maximized log-likelihood, assuming the logistic regression model holds. Chen and Chen (2004) also propose a frequentist asymptotic goodness of fit test in the context of case-control studies. It is shown here that a Bayesian goodness of fit test arises very naturally and that it has a number of advantages. In particular, evidence can be obtained in favor of the logistic regression model, as opposed to only evidence against as with *p*-values, and there is no appeal to asymptotics.

## The Model and prior

Supposed there are *m* observations *s*(**X**)=(*s*(**x**_1_),…,*s*(**x**_*m*_)) where *s*(**x**_*i*_)∼ binomial (*n*(**x**_*i*_),*θ*(**x**_*i*_)), the *s*(**x**_*i*_) are independent, *θ*(**X**)=(*θ*(**x**_1_),…,*θ*(**x**_*m*_))^′^∈[0,1]^*m*^ and **X**=(**x**_1_,…,**x**_*m*_)^′^∈*R* ^*m*×*k*^. Intuitively, a noninformative prior for the *θ*(**X**_*m*_) is then given by the uniform distribution on [0,1]^*m*^ as this allows for any of the possible values and they are all weighted equally. Of course, other definitions can be provided for noninformativity but this definition seems most suitable for this context as it possesses a key property.

To see this suppose that we have a statistical model {*f*_*θ*_:*θ*∈*Θ*} for the data *x*, and a prior *π*. Let *T* denote a minimal sufficient statistic for the model with marginal model {*f*_*θ*,*T*_:*θ*∈*Θ*}. Then $m_{T}(t)=\int _{\Theta }f_{\theta,T}(t)\,\Pi (d\theta)$ is the prior predictive density of *T*. In Evans and Moshonov (2006) a basic check on the prior is to compute the tail probability *M*_*T*_(*m*_*T*_(*t*)≤*m*_*T*_(*T*(*x*))) and conclude that prior-data conflict exists whenever this probability is small as this implies that the observed data is a priori surprising. The consistency of this check, under quite general conditions, is established in Evans and Jang (2011). If, for example, *M*_*T*_(*m*_*T*_(*t*)≤*m*_*T*_(*T*(*x*)))≡1 for all possible *T*(*x*), then it is never the case that there is prior-data conflict and the prior can be called noninformative. Clearly this criterion for noninformativity can be weakened but this is all that is required here.

For the product-binomial, *T* can be taken equal to *s*(**X**). Since the counts are independent and the priors on the *θ*(**x**_*i*_) are independent and uniform, the prior predictives of the *s*(**x**_*i*_) are independent. The prior predictive for *s*(**x**_*i*_) is easily seen to equal 1/(*n*(**x**_*i*_)+1), namely, it is uniform on {0,1,…,*n*(**x**_*i*_)}. As such, for the product-binomial with a uniform prior, *M*_*T*_(*m*_*T*_(*t*)≤*m*_*T*_(*T*(*x*)))≡1 and our criterion for noninformativity is satisfied. The posterior on *θ*(**X**) induced by this prior and the observed data *s*(**X**) is a product of beta distributions where *θ*(**x**_*i*_) | *s*(**x**_*i*_)∼ beta (*s*(**x**_*i*_)+1,*n*(**x**_*i*_)−*s*(**x**_*i*_)+1). Note that is easy to generate from both the prior and posterior of *θ*(**X**).

## Hypothesis assessment via relative belief and concentration

Suppose for the model {*f*_*θ*_:*θ*∈*Θ*} it is desired to assess the hypothesis *H*_0_⊂*Θ*. In many cases there is a parameter of interest *ψ*=*Ψ*(*θ*) with *H*_0_=*Ψ* ^−1^{*ψ*_0_}. The evidence for or against *H*_0_ can then be assessed via a relative belief ratio *R* *B*_*Ψ*_(*ψ*_0_ | *x*)=*π*_*Ψ*_(*ψ*_0_ | *x*)/*π*_*Ψ*_(*ψ*_0_), the ratio of the posterior to prior densities of *Ψ* evaluated at the hypothesized value. If *R* *B*_*Ψ*_(*ψ*_0_ | *x*)>1, then there is evidence in favor of *H*_0_, as the posterior probability for *ψ*_0_ is greater than the prior probability for *ψ*_0_, while *R* *B*_*Ψ*_(*ψ*_0_ | *x*)<1 implies there is evidence against *H*_0_ and when *R* *B*_*Ψ*_(*ψ*_0_ | *x*)=1 there isn’t evidence either way. The strength of the evidence is measured by the posterior probability

2 $$ \Pi_{\Psi}(RB_{\Psi}(\psi\,|\,x)\leq RB_{\Psi}(\psi_{0}\,|\,x)\,|\,x).  $$

For when *R* *B*_*Ψ*_(*ψ*_0_ | *x*)<1 and (2) is small, then there is a strong belief that the true value of *Ψ* has a larger relative belief ratio than *ψ*_0_ and so the evidence against *ψ*_0_ is strong. When *R* *B*_*Ψ*_(*ψ*_0_ | *x*)<1 and (2) is large, then there is only weak evidence against *H*_0_ as this says that there is a large belief that the true value of *ψ* has the value of its relative belief ratio no greater than *R* *B*_*Ψ*_(*ψ*_0_ | *x*). When *R* *B*_*Ψ*_(*ψ*_0_ | *x*)>1 and (2) is large, then there is a weak belief that the true value of *Ψ* has a larger relative belief ratio than *ψ*_0_ and so the evidence in favor of *ψ*_0_ is strong. Note that in the set {*ψ*:*R* *B*_*Ψ*_(*ψ* | *x*)≤*R* *B*_*Ψ*_(*ψ*_0_ | *x*)} the value *ψ*_0_ has the most evidence in its favor when *R* *B*_*Ψ*_(*ψ*_0_ | *x*)>1. When *R* *B*_*Ψ*_(*ψ*_0_ | *x*)>1 and (2) is small, then there is only weak evidence in favor *H*_0_ as this says that there is a large belief that the true value of *ψ* has the value of its relative belief ratio greater than *R* *B*_*Ψ*_(*ψ*_0_ | *x*). The relative belief ratio is discussed in Baskurt and Evans (2013) and a full development of a theory of inference based on this is presented in Evans (2015).

The interpretation of the relative belief ratio as the evidence demands that a relative belief ratio greater than 1 be interpreted as evidence in favour of the hypothesis no matter how much greater it is than 1, assuming it is computed exactly. This is because *R* *B*_*Ψ*_(*ψ*_0_ | *x*)>1 occurs only when the posterior probability is greater than the prior probability of the hypothesis and that is the basic criterion for saying the data has led to evidence in favor. A similar comment applies to evidence against, namely, when *R* *B*_*Ψ*_(*ψ*_0_ | *x*)<1. To understand what *R* *B*_*Ψ*_(*ψ*_0_ | *x*)=1 means consider a discrete context as then this occurs iff the posterior probability of {*ψ*_0_} equals the prior probability of {*ψ*_0_} and this occurs iff the events {*ψ*_0_} and {*x*} are statistically independent in the joint probability model for (*θ*,*x*). In other words *R* *B*_*Ψ*_(*ψ*_0_ | *x*)=1 iff the actual observed data *x* tells us nothing about the hypothesis that the true value of *ψ*=*Ψ*(*θ*) is *ψ*_0_. This is clearly a very unusual circumstance but one can easily construct such situations generally when the model contains nonidentifiability. In essence the relative belief ratio is giving the correct assessment of evidence in such a case.

The size of *R* *B*_*Ψ*_(*ψ*_0_ | *x*) does not necessarily reflect the strength of the evidence. Note that in the discrete case *R* *B*_*Ψ*_(*ψ*_0_ | *x*)=*π*_*Ψ*_(*ψ*_0_ | *x*)/*π*_*Ψ*_(*ψ*_0_)≤1/*π*_*Ψ*_(*ψ*_0_) so there is an upper bound on this value. From this it is seen that relative belief ratios do not measure evidence on an absolute scale but they need to be calibrated in each context and that is the role of the strength (2). So even if *R* *B*_*Ψ*_(*ψ*_0_ | *x*)=1.000005 the strength could be high when (2) is close to 1 as this says our belief that the true value of *ψ* has a larger relative belief ratio is small. Note that the strength is playing the role of the standard error here as it measures how reliable we believe our assessment of the evidence is. Also, it can happen that even though *R* *B*_*Ψ*_(*ψ*_0_ | *x*) is very high, (2) can be very small and so the evidence is only weak evidence in favor. This phenomenon is associated with the Jeffreys-Lindley paradox as is discussed in Evans (2015). In short, relative belief ratios need to be calibrated and the calibration depends on the context. The issues concerning measuring strength are a somewhat more involved than the measure of the evidence itself and additional discussion can be found in Evans (2015).

In a number of situations *H*_0_ does not arise via *H*_0_=*Ψ* ^−1^{*ψ*_0_} for some *Ψ* in an obvious way and also *Π*(*H*_0_)=0. The prior nullity may arise because *H*_0_ is a lower dimensional subset of *Θ* and not because there is no belief that *H*_0_ is true. This is the case with logistic regression when *k*<*m* and *Π* is the uniform prior on *Θ*=[0,1]^*m*^. In such a context it is reasonable to choose $\Psi =d_{H_{0}}$ where $d_{H_{0}}(\theta (\mathbf {X}))$ is a measure of the distance of *θ*(**X**) to *H*_0_. So with *ψ*_0_=0 then *H*_0_=*Ψ* ^−1^{*ψ*_0_} and *H*_0_ can be assessed using relative belief. Note that it is clear that in assessing *H*_0_ a comparison is being made between the concentrations of the prior and posterior about *H*_0_. If *R* *B*_*Ψ*_(*ψ*_0_ | *x*)>1, then the data has lead to the posterior being more concentrated about *H*_0_ than the prior. If *R* *B*_*Ψ*_(*ψ*_0_ | *x*)<1, then the data has lead to the posterior being less concentrated about *H*_0_ than the prior. The method of concentration, with $d_{H_{0}}$ equal to squared Euclidean distance as discussed in Example 1, was developed for some specific inference problems in Evans et al. (1993, 1997).

While there are many possible choices for $d_{H_{0}},$ two are considered here.

### **Example 1**

*Squared Euclidean distance.* Let *μ*(**x**)= log(*θ*(**x**)/(1−*θ*(**x**))) denote the logit associated with **x** and note that the logistic regression model holds iff *μ*(**x**)=**x** ^′^ **β** for some **β**∈*R* ^*k*^ for every **x**∈*R* ^*k*^. The logistic regression model thus implies that *μ*(**X**)=**X** **β** for some **β**∈*R* ^*k*^. If a probability distribution is placed on *θ*(**X**), then this induces a probability distribution on *μ*(**X**) which in turn induces a probability distribution on $d_{H_{0}}(\theta (\mathbf {X}))=\inf _{\mathbf {\beta }\in R^{k}}||\mu (\mathbf {X})-\mathbf {X\beta }||^{2}/m=\mu (\mathbf {X})^{t}(I-\mathbf {X} (\mathbf {X}^{t}\mathbf {X})^{-1}\mathbf {X}^{t})\mu (\mathbf {X})/m$ where it is assumed that **X** is of full rank. The reason for dividing by the dimension *m* of *μ*(**X**) will become apparent in Example 3 although this clearly has no effect on the optimization. Note that $d_{H_{0}} (\theta (\mathbf {X}))=0$ iff the logistic regression model holds for the observed *s*(**X** **)**. So it is natural to measure the concentration of the probability distribution placed on *θ*(**X**) about *H*_0_ by seeing how concentrated the induced distribution on $d_{H_{0}}(\theta (\mathbf {X}))$ is about 0.

### **Example 2**

*Kullback-Leibler (KL) distance.* The KL distance between the Bernoulli (*θ*)and the Bernoulli (*p*)distribution is given by *K* *L*(*θ*,*p*)=*θ* log(*θ*/*p*)+(1−*θ*) log((1−*θ*)/(1−*p*)). It follows that the KL distance between the product-Bernoulli (*θ*_1_,…,*θ*_*m*_) and the product-Bernoulli (*p*_1_,…,*p*_*m*_) equals ${\sum \nolimits }_{i=1}^{m}KL(\theta _{i},p_{i}).$ So we put

$$d_{H_{0}}(\theta(\mathbf{X}))=\inf_{\mathbf{\beta\in}R^{k}}\sum\limits_{i=1} ^{m}KL(\theta(\mathbf{x}_{i}),p_{i}\mathbf{(\mathbf{x}^{\prime}\mathbf{\beta })})/m $$

and the reason for dividing by *m* is discussed in Example 4. Given an efficient algorithm for finding the optimal **β** **,** it is straightforward to generate from the prior and posterior distributions of $d_{H_{0}}(\theta (\mathbf {X})).$

After a choice is made of $d_{H_{0}},$ the methodology proceeds via simulation from the prior and posterior distributions of $d_{H_{0}}(\theta (\mathbf {X}))$, then computing $RB_{d_{H_{0}}} (0\,|\,s(\mathbf {X}))$ and its strength. A significant aspect of this computation is that the prior and posterior densities of $d_{H_{0}}$ typically both vanish at 0 and so the ratio cannot be directly computed. This is a support measure issue arising due to continuity and is dealt with theoretically by defining the relative belief ratio at a point as the limiting ratio of the posterior to prior probabilities of shrinking neighborhoods. Practically this is dealt with by the choice of *δ* since $d_{H_{0} }(p)\in [0,\delta)$ implies that *H*_0_ holds to the accuracy required in the application. In other words, *H*_0_ holds whenever the difference between the true model and the logistic regression model is of no practical consequence as measured by $d_{H_{0}}$. The range of the prior distribution of $d_{H_{0}}$ is then discretized via the partition {[0,*δ*),[*δ*,2*δ*),…,[(*k*−1)*δ*,*k* *δ*)} where *k* is chosen so that the effective range of this prior distribution is covered. The prior and posterior probability contents of these intervals are estimated by generating large samples from the prior and the posterior distributions of *θ*, computing $d_{H_{0}}(\theta)$ for each sampled value, which gives samples from the prior and posterior distributions of $d_{H_{0}}(\theta),$ and then using the approximate contents for the approximation of the relative belief ratios of the intervals. The relevant relative belief ratio for assessing *H*_0_ is then $RB_{d_{H_{0}}}([0,\delta)\,|\,s(\mathbf {X}))$ and the strength of this evidence is assessed by comparing this relative belief ratio against the other values by computing the posterior probability that $RB_{d_{H_{0}}} ([i\delta,(i+1)\delta)\,|\,s(\mathbf {X}))\leq RB_{d_{H_{0}}}([0,\delta)\,|\,s(\mathbf {X}))$ where the Monte Carlo estimates were used for these computations. It is proved in Evans (2015) that this procedure is consistent as the amount of data increases in the sense that the relative belief ratio converges to the maximum possible value (always greater than 1) and the strength converges to 1 when *H*_0_ is true, and the relative belief ratio converges to 0 and the strength converges to 0 when *H*_0_ is false.

The choice of *δ* is application dependent as it represents the deviation from the precise null that is just of practical consequence. The two distance measures considered here lead to very natural choices for *δ*.

### **Example 3**

*Squared Euclidean distance and absolute error.* For this distance measure let *δ* equal the maximum squared distance between two logits such that any difference smaller than *δ* is practically speaking immaterial. In other words, if $\max _{i}(\log (\theta (\mathbf {x}_{i})/(1-\theta (\mathbf {x}_{i})))-\mathbf {x}_{i}^{\prime }\mathbf {\beta })^{2}<\delta,$ then this difference is irrelevant from the point of view of the application. It is clear then that $(\mu _{i} (\mathbf {X})-\mathbf {x}_{i}^{\prime }\mathbf {\beta)}^{2}\mathbf {<\,}\delta $ for *i*=1,…,*m* implies ||*μ*(**X**)−**X** **β**||^2^/*m* **<** *δ* while ||*μ*(**X**)−**X** **β**||^2^/*m* **<** *δ* implies that the average squared absolute error between individual logits is less than *δ*. So in practice we proceed by selecting *δ* and discretizing the prior and posterior distributions of $d_{H_{0}}$ as previously described. Note that it is also reasonable to choose a discretization parameter *δ*_∗_<*δ* for $d_{H_{0}}.$ For example, if *δ*_∗_=*δ*/*m* then ||*μ*(**X**)−**X** **β**||^2^/*m*< *δ*_∗_ implies $\max _{i}(\mu _{i}(\mathbf {X})-\mathbf {x}_{i}^{\prime }\mathbf {\beta)}^{2}\mathbf {<\,}\delta $ but this might be deemed overly rigorous.

While the interpretation of error in the value of **x** ^′^ **β** is straightforward in linear regression, this is more difficult in logistic regression and it then seems clearer to state bounds on the probabilities. Note, however, for probabilities *θ* and *p*, the logits satisfy (log(*θ*/(1−*θ*))− log(*p*/(1−*p*)))^2^=(log(*θ*(1−*p*)/*p*(1−*θ*)))^2^<*δ* iff exp(−*δ* ^1/2^)<1+(*θ*−*p*)/*p*(1−*θ*)< exp(*δ* ^1/2^), and using *e* ^*x*^≈1+*x* for small *x*, this is approximately equivalent to (*θ*−*p*)^2^<*p* ^2^(1−*θ*)^2^ *δ*≤*δ* when *δ* is small. So, if *δ* is chosen to reflect what is considered a meaningful absolute squared difference in the probabilities, then the logits satisfying this error bound implies that the probabilities also satisfy this, at least when *δ* is small.

### **Example 4**

*Kullback-Leibler (KL) distance and relative error.* For this distance measure let *δ* equal the maximum relative error in the probabilities. So it is desired that max*i*|(*θ*_*i*_−*p*_*i*_)/*θ*_*i*_|<*δ* and max*i*|((1−*θ*_*i*_)−(1−*p*_*i*_))/(1−*θ*_*i*_)|<*δ*. These inequalities hold iff − log(1+*δ*)< log(*θ*_*i*_/*p*_*i*_)<− log(1−*δ*) and − log(1+*δ*)< log((1−*θ*_*i*_)−(1−*p*_*i*_))/(1−*θ*_*i*_)<− log(1−*δ*) for every *i*, which implies $-\log (1+\delta)<{\sum \nolimits }_{i=1}^{m}KL(\theta _{i},p_{i})/m<-\log (1-\delta)$ and the lower bound can be replaced by 0 since the KL distance is always nonnegative. Using log(1+*x*)≈*x* when *x* is small, a small relative error of *δ* on the probabilities then implies the approximate bounds $0\leq {\sum \nolimits }_{i=1}^{m}KL(\theta _{i},p_{i})/m<\delta.$ Conversely, ${\sum \nolimits }_{i=1}^{m}KL(\theta _{i},p_{i})/m<\delta $ implies that the average relative error in the probabilities is bounded by *δ*. This gives the discretization for the prior and posterior distributions for $d_{H_{0}} (\theta (\mathbf {X}))$ in this case. Again a discretization parameter *δ*_∗_<*δ* can be used for $d_{H_{0}}(\theta (\mathbf {X}))$ if a bound on the average relative error on the individual probabilities is not felt to be rigorous enough.

It is emphasized that the choice of the distance measure and the discretization parameter are application dependent. Given that the concern is with model checking, and there are often many ways in which a model can be checked, the choice of the distance measure is perhaps not important. On the other hand, when choosing between the distance measures suggested here, this could be determined by the choice of absolute or relative error as the criterion of accuracy. When the probabilities in question are not too small or too large, then absolute error seems like the appropriate error criterion to use, and hence use squared Euclidean distance, while when probabilities are felt to be close to 0 or 1, then relative error seems like the appropriate error criterion and so use Kullback-Liebler distance.

Some may object to the need to discretize. In our view the choice of a *δ* to specify practically relevant deviations is a necessary aspect of any meaningful inference problem. It seems realistic to say that a logistic regression model is never strictly correct as there is no reason to suppose that the probabilities are exactly given by (1) for any **x**. What is more relevant is whether or not the logistic regression model is approximately correct and to make the notion of approximation precise one has to specify a *δ*. For example, if a logistic regression model provided two or three decimal accuracy for the relevant probabilities, then it could be that this is sufficient accuracy but this depends on the application as sometimes greater accuracy may be required. Examples 3 and 4 provide prescriptions for how *δ* can be chosen to reflect the accuracy desired in a problem. It would seem very odd that an individual familiar with the application couldn’t specify such an accuracy as it cannot be true that any deviation whatsoever is significant as this contradicts the approximate nature of the logistic regression model. Provided the prior distribution is relatively smooth, as is the case here, the results will not change much by making small changes in *δ*, as changes in the prior and posterior probabilities will also be small. Also, as is well known, *p*-values can detect deviations from hypotheses that are not practically meaningful when sample sizes are large. The way to avoid this behavior is to build the relevant deviation directly into the inference methodology and that is what is done here.

## Examples

Implementation of the computations is relatively straight-forward via simulation once $d_{H_{0}}$ and *δ* have been selected, although clearly using squared Euclidean distance is somewhat easier. For the optimization with KL distance, the R routine optim was used. In all the examples the prior and posterior distributions of $d_{H_{0}}$ were approximated using a Monte Carlo sample of size of 10^5^ and these distributions were then discretized as previously discussed.

Some simulated examples are now considered where each distance is applied when the logistic regression model holds and when it doesn’t.

### **Example 5**

*Simulated examples when logistic regression is correct.* Consider the situation where *k*=2 with *X*_1_≡1 and *X*_2_ is a nonconstant quantitative predictor, so *p*(**x** ^′^ **β**)= exp{*β*_1_+*β*_2_ *x*_2_}/(1+ exp{*β*_1_+*β*_2_ *x*_2_}). Various choices are considered for *n*=*n*(**x**_1_)=⋯=*n*(**x**_*m*_) and for *δ*, the squared absolute error in the respective probabilities when using Euclidean distance and the relative error in the respective probabilities when using KL distance. Note that in practice *δ* and the *n*(**x**_*i*_) are fixed in an application. Here *m*=3 with *x*_2_∈{0,1,2} and *β*_1_=0.5,*β*_2_=−1.0 so *p*(**X**)=(0.62,0.38,0.18) gives the true probabilities for the corresponding Bernoulli distributions.

Table 1 gives the results of some simulations when using squared Euclidean distance as the basis for the measure of concentration. When *n*=1 the data *s*(**X**)=(1,0,0) was obtained, when *n*=5 the data *s*(**X**)=(4,2,1) was obtained and when *n*=10 the data *s*(**X**)=(7,6,1) was obtained. Notice that the relative belief ratio is always greater than 1 which says there is evidence in favour of the logistic regression model being true. The strength of this evidence depends on *δ* but not greatly and it is to be noted that this is determined by how much squared error is acceptable in the probabilities provided by the model. The *p*-value based on the deviance gave the values 1.0, 0.74, 0.22 when *n*=1,5,10 respectively, and so this test would also not reject the logistic regression model in these simulations. It is to be noted, however, that the *p*-value is asymptotic and it is not clear how large *n* has to be for this approximation to be accurate. Also, in contrast to the relative belief ratio, a large *p*-value does not provide evidence in favor of *H*_0_. It is of interest that, while it is expected that both the relative belief ratio and its strength will increase as *n* increases, this did not happen when comparing *n*=5 with *n*=10. This is undoubtedly due to sampling variability as there is no guarantee that increasing sample size increases accuracy.

**Table 1:** The values of *RB* together with the (strength) of the evidence in Example 5 when *m*=3 using squared Euclidean distance. The effective range of the prior is [0,4.0)

*δ*	*n*=1	*n*=5	*n*=10
0.001	1.05(0.46)	1.99(0.89)	1.43(0.46)
0.010	1.05(0.52)	1.98(1.00)	1.43(0.46)
0.050	1.07(0.92)	1.91(1.00)	1.46(0.73)
0.100	1.07(0.92)	1.85(1.00)	1.46(0.73)

Table 2 gives the results of the analysis of the data when using KL distance as the basis for the measure of concentration. Again the relative belief ratio is always greater than 1 and so gives the correct inference. The strength of the evidence in favor is always at least as great as when using squared Euclidean distance. This underscores a reasonable conjecture that KL distance is a more appropriate measure of concentration in this problem than squared Euclidean distance.

**Table 2:** The values of *RB* together with the (strength) of the evidence in Example 5 when *m*=3 using KL distance. The effective range of the prior is [0,0.4)

*δ*	*n*=1	*n*=5	*n*=10
0.001	1.07(0.73)	1.71(0.96)	1.29(0.42)
0.010	1.06(1.00)	1.67(1.00)	1.32(1.00)
0.050	1.06(1.00)	1.45(1.00)	1.36(1.00)
0.100	1.05(1.00)	1.27(1.00)	1.26(1.00)

To investigate the effect of increasing the number of values of the predictor, consider a simulated example where *k*=2,*m*=20 with

$$\begin{array}{*{20}l} x_{2} & \in \{-1.35,-1.32,-0.87,-0.77,-0.59,-0.56,-0.44,-0.34,-0.23,\\ & -0.15,-0.02,0.016,0.05,0.17,0.42,0.68,1.10,1.15,1.80,2.01\}. \end{array} $$

Setting *β*_1_=0.5,*β*_2_=1.0 leads to the following probabilities for the corresponding Bernoulli distributions

$$\begin{array}{*{20}l} p(\mathbf{X}) &=(0.206,0.210,0.296,0.316,0.356,0.364,0.392,0.417,0.443,0.463,\\ &\quad 0.494,0.504,0.513,0.543,0.604,0.663,0.750,0.760,0.858,0.882). \end{array} $$

When *n*=1, *s*(**X**)=(0,0,0,0,1,0,0,0,0,0,1,0,0,1,0,1,1,0,1,1), when *n*=5, *s*(**X**)=(0,0,3,2,3,2,3,1,3,2,1,3,1,1,4,3,2,3,3,5) and when *n*=10, *s*(**X**)=(1,3,4,3,2,6,4,5,3,0,7,7,8,7,6,3,8,8,8,7) are the corresponding generated data sets. Table 3 gives the results when using squared Euclidean distance and Table 4 gives the results when using KL distance, respectively, as the basis for the measure of concentration. Notice that the relative belief ratio is always greater than 1 which says correctly that there is evidence in favour of the logistic regression model being true. The strength of this evidence depends on *δ* and it is to be noted that this is determined by how much squared error is acceptable in the probabilities provided by the model. Of some interest is the fact that the strength drops in this example as *δ* increases and this is undoubtedly due to the relative belief ratio $RB_{d_{H_{0}}} ([0,\delta)\,|\,s(\mathbf {X}))$ decreasing as greater tolerance is permitted for the approximation given by the logistic regression model. The posterior probability of the interval [0,*δ*) increases with *δ* but for this dataset, it does not increase as fast as the prior probability so the evidence, as measured by change in belief, is not as great for larger values of *δ*. This underlines the importance of choosing *δ* to reflect desired accuracy. The *p*-value based on the deviance gave the values 0.40,0.22,0.01 when *n*=1,5,10 respectively. So in fact the deviance test leads to a *p*-value that would indicate that the model is not true when *n*=10 and this is incorrect.

**Table 3:** The values of *RB* together with the (strength) of the evidence in Example 5 when *m*=20 using squared Euclidean distance. The effective range of the prior is [0,12.0)

*δ*	*n*=1	*n*=5	*n*=10
0.001	2.43(1.00)	69.63(1.00)	38.47(1.00)
0.010	1.86(1.00)	28.77(1.00)	24.51(1.00)
0.050	1.61(1.00)	12.07(0.40)	12.03(0.39)
0.100	1.50(0.92)	7.61(0.40)	7.66(0.39)

**Table 4:** The values of *RB* together with the (strength) of the evidence in Example 5 when *m*=20 using KL distance. The effective range of the prior is [0,0.35)

*δ*	*n*=1	*n*=5	*n*=10
0.001	2.20(1.00)	53.00(1.00)	39.90(1.00)
0.010	2.15(1.00)	31.50(1.00)	30.10(1.00)
0.050	1.88(1.00)	13.50(0.32)	13.98(0.30)
0.100	1.76(0.91)	8.16(0.32)	8.55(0.30)

### **Example 6**

*Simulated examples when logistic regression is not correct.* Simulated examples are now considered when the logistic regression model with **x**=(1,*x*_2_)^′^ is wrong. Here *m*=5 and the values *x*_2_∈{1,3,5,7,9} were chosen with the true probabilities given by *θ*(**X**)=(0.875,0.327,0.107,0.198,0.908). The average squared Euclidean distance between these product-Bernoulli probabilities and the best fitting logistic regression with the corresponding values for *x*_2_ is 0.117, so the logistic regression model is definitely false. The following data sets were generated from the true model: when *n*=1, then *s*(**X**)=(1,0,0,0,1), when *n*=5 then *s*(**X**)=(5,2,0,1,5) and when *n*=10 then *s*(**X**)=(9,3,1,2,9).

Table 5 records the results of the goodness of fit test based on squared Euclidean distance. In every case the relative belief ratio is less than 1, so there is evidence against *H*_0_, and the strength of this evidence is strong. The *p*-values based on the deviance statistic are respectively 0.08,0.00,0.00. So, excepting the *n*=1 case, this approach also clearly rejects *H*_0_. Table 6 records the results of the goodness of fit test based on KL distance. In every case the relative belief ratio is less than 1 and the strength of this evidence is definitive with the possible exception of two cases when *n*=1.

**Table 5:** The values of *RB* together with the (strength) of the evidence in Example 6 when *m*=5 using squared Euclidean distance. The effective range of the prior is [0,3.0)

*δ*	*n*=1	*n*=5	*n*=10
0.001	0.00(0.00)	0.00(0.00)	0.00(0.00)
0.010	0.38(0.00)	0.00(0.00)	0.00(0.00)
0.050	0.66(0.00)	0.00(0.00)	0.00(0.00)
0.100	0.68(0.01)	0.00(0.00)	0.00(0.00)

**Table 6:** The values of *RB* together with the (strength) of the evidence in Example 6 when *m*=5 using KL distance. The effective range of the prior is [0,0.3)

*δ*	*n*=1	*n*=5	*n*=10
0.001	0.55(0.00)	0.00(0.00)	0.00(0.00)
0.010	0.61(0.01)	0.00(0.00)	0.00(0.00)
0.050	0.69(0.14)	0.02(0.00)	0.01(0.00)
0.100	0.78(0.36)	0.09(0.04)	0.08(0.04)

Again the case *m*=20 is considered with *x*_2_ taking values as in Example 5 and the true probabilities, obtained using a logistic regression containing a quadratic term, given by

$$\begin{array}{*{20}l} \theta(\mathbf{X}) &= (0.0003, 0.0004, 0.0582, 0.1230, 0.3229, 0.3617, 0.5080, 0.6040, 0.6760, \\ &\quad\; 0.7084, 0.7307, 0.7308, 0.7286, 0.7017, 0.5295, 0.2122, 0.0064, 0.0036,\\ &\quad\; 0.0000, 0.0000). \end{array} $$

The average squared Euclidean distance between these product-Bernoulli probabilities and the best fitting linear logistic regression with the corresponding values for *x*_2_ is 0.231, so the linear logistic regression model is definitely false. The following data sets were generated from the true model: when *n*=1 then *s*(**X**)=(1,1,0,0,0,0,0,1,1,0,0,1,1,0,1,1,1,0,1,1), when *n*=5 then *s*(**X**)=(5,4,5,1,1,3,0,1,2,1,1,0,2,4,5,3,4,4,5,4) and when *n*=10 then *s*(**X**)=(8,7,10,1,2,2,4,5,1,1,1,0,6,8,7,7,6,10,10,10) were obtained. Table 7 records the results when using squared Euclidean distance and Table 8 gives the results when using KL distance. It is to be noted that in every case there is strong evidence against the (linear) logistic model holding and the results are robust to the choice of *δ*. The *p*-values based on the deviance statistic are respectively 0.08,0.00,0.00 so, as might be expected, the test does not do as well when *n*=1.

**Table 7:** The values of *RB* together with the (strength) of the evidence in Example 6 when *m*=20 using squared Euclidean distance. The effective range of the prior is [0,15.0)

*δ*	*n*=1	*n*=5	*n*=10
0.001	0.68(0.00)	0.11(0.00)	0.00(0.00)
0.010	0.77(0.00)	0.22(0.00)	0.01(0.00)
0.050	0.87(0.04)	0.42(0.00)	0.06(0.00)
0.100	0.87(0.04)	0.54(0.02)	0.13(0.00)

**Table 8:** The values of *RB* together with the (strength) of the evidence in Example 6 when *m*=20 using KL distance. The effective range of the prior is [0,0.3)

*δ*	*n*=1	*n*=5	*n*=10
0.001	0.60(0.00)	0.20(0.00)	0.00(0.00)
0.010	0.54(0.00)	0.24(0.00)	0.01(0.00)
0.050	0.78(0.04)	0.44(0.04)	0.06(0.00)
0.100	0.81(0.04)	0.50(0.04)	0.14(0.00)

The following example presents an application to a real data set.

### **Example 7**

*Bioassay experiment.* In studying drugs and other chemical compounds, acute toxicity tests or bioassay experiments are commonly implemented on animals. Such experiments proceed by controlling various dose levels of the compound to groups of animals. The animals responses are typically characterized as alive or dead, tumor or no tumor, etc. The logistic regression model with **x**=(1,*x*_2_)^′^ is considered where *x*_2_ is the log of the dosage in g/ml of a toxin. The data is provided in Table 9 and comes from a real data set analyzed in Racine et al. *(* 1986 *)* where *m*=4,*n*(**x**_1_)=⋯=*n*(**x**_4_)=5 and *s*(**x**_*i*_) is the number of deaths at the *i*-th dosage.

**Table 9:** Data in Example 7

*x*_2_	No. of animals	No. of deaths
−0.86	5	0
−0.30	5	1
−0.05	5	3
0.73	5	5

Since the authors were not part of this application the value of *δ* cannot be strictly determined by what the goals of the study are. As such, the results are considered for a fairly wide range of possible values for *δ* in Table 10 and it is seen that the results are robust to this choice. In all cases the relative belief ratios show that there is evidence in favor of the logistic regression model holding and the strength of this evidence is universally very strong especially when using KL distance. The *p*-value based on deviance is 0.97 so the logistic regression model is not rejected but again it is to be noted that, following the logic of *p*-values, this is not to be interpreted as support for this model.

**Table 10:** The values of *RB* together with the (strength) of the evidence in Example 7 using squared Euclidean distance (effective range of the prior of $d_{H_{0}}$ is [0,3.0)) and KL distance (the effective range of the prior of $d_{H_{0}}$ is [0,0.3))

*δ*	Squared Euclidean distance	KL distance
0.001	2.67(0.90)	3.53(1.00)
0.010	2.67(0.97)	3.13(1.00)
0.050	2.55(0.99)	2.20(1.00)
0.100	2.47(0.99)	1.61(1.00)

## Conclusions

A Bayesian goodness of fit test has been developed for logistic regression models based on a measure of evidence. A definite advantage of this approach is that evidence can be obtained in favor of the model holding. Also, there is no need to appeal to asymptotics in the interpretation of the results as in the case of classical goodness of fit tests. Since every product-Bernoulli distribution is treated equally in the priors there is no bias towards accepting or rejecting the logistic regression model. The choice of which distance measure to use is dependent on whether relative or absolute error is the appropriate criterion to apply when considering the approximation a logistic regression model supplies to the true probabilities. The approach developed in this paper can also be used for goodness of fit tests for other models such as probit regression with only minor changes.
